# In Situ Control of Thermal Activation Conditions by Color for Serpentines with a High Iron Content

**DOI:** 10.3390/ma14216731

**Published:** 2021-11-08

**Authors:** Tatiana K. Ivanova, Irina P. Kremenetskaya, Andrey I. Novikov, Valentin G. Semenov, Anatoly G. Nikolaev, Marina V. Slukovskaya

**Affiliations:** 1 I.V. Tananaev Institute of Chemistry and Technology of Rare Elements and Mineral Raw Materials, Kola Science Centre, Russian Academy of Sciences, 184209 Apatity, Russia; tk.ivanova@ksc.ru (T.K.I.); i.kremenetskaia@ksc.ru (I.P.K.); a.novikov@ksc.ru (A.I.N.); 2 Laboratory of Nature-Inspired Technologies and Environmental Safety of the Arctic, Kola Science Centre, Russian Academy of Sciences, 184209 Apatity, Russia; 3 Institute for Analytical Instrumentation, Russian Academy of Sciences, 198095 St. Petersburg, Russia; val_sem@mail.ru; 4 Department of Mineralogy and Lithology, Institute of Geology and Petroleum Technologies, Kazan Federal University, 420008 Kazan, Russia; anatolij-nikolaev@yandex.ru; 5 Department of Landscape Design and Sustainable Ecosystems, RUDN University, 117198 Moscow, Russia

**Keywords:** serpentine, chrysotile, iron, muffle furnace, rotary furnace, X-ray diffraction, Mössbauer spectroscopy, optical spectroscopy, color models, color difference

## Abstract

Serpentine heat treatment at temperatures of 650–750 °C yields magnesium–silicate reagent with high chemical activity. Precise and express control of roasting conditions in laboratory kilns and industrial aggregates is needed to derive thermally activated serpentines on a large scale. Color change in serpentines with a high iron content during roasting might be used to indicate the changes in chemical activity in the technological process. This study gives a scientific basis for the express control of roasting of such serpentines by comparing the colors of the obtained material and the reference sample. Serpentines with different chemical activity were studied by X-ray diffraction, Mössbauer spectroscopy, and optical spectroscopy. The color parameters were determined using RGB (red, green, blue), CIELAB (International Commission on Illumination 1976 L*a*b), and HSB (hue, brightness, saturation) color models. The color of heat-treated samples was found to be affected by changes in the crystallochemical characteristics of iron included in the structure of the serpentine minerals. The color characteristics given by the CIELAB model were in good coherence with the acid-neutralizing ability and optical spectra of heat-treated serpentines. Thus, in contrast to the long-term analysis by these methods, the control by color palette provides an express assessment of the quality of the resulting product.

## 1. Introduction

Serpentine minerals are widespread in the Earth’s crust, including the overburden and host rocks of deposits of magnesian raw materials such as magnesite, olivenite, phlogopite, etc. Serpentine-containing wastes stored in dumps may pose environmental problems and need to be disposed of [1,2]. Simultaneously, serpentinites are of interest as a raw material source for obtaining various products based on magnesium, silicon, iron, nickel, and chromium oxides.

Serpentine minerals belong to the hydrous magnesium group silicates and have the chemical formula 3MgO_2_·2SiO_2_·2H_2_O. There are three main crystalline modifications of serpentine minerals: antigorite, lizardite, and chrysotile [3]. In addition, isomorphic substitutions of magnesium and silicon are possible, and the general formula can be represented as Mg_3-x_(M)_x_Si_2-y_(T)_y_O_5_(OH)_4_, where M-Mg^2+^, Fe^2+^, Fe^3+^, Al^3+^, Ni^2+^, Mn^2+^, Zn^2+^, and T-Si^4+^, Al^3+^, Fe^3+^ [4]. The use of serpentinites is problematic due to the wide variety of their properties, which hinders development of effective technologies. However, practically unlimited reserves of serpentines, including mining waste, stimulate the search for new ways to use them.

The series of studies carried out under the leadership of N.O. Zulumyan should be noted [5,6,7]. This research group developed a thermal-acid treatment method of serpentines based on their ability to form an active metastable magnesium silicate phase under heat treatment [7,8]. Thermally activated serpentines obtained by this method were proposed as an alkaline magnesium silicate reagent for cleaning of aqueous solutions from metals [9] and remediation of contaminated soils. Serpentine heat treatment at 650–750 °C makes it possible to obtain a reagent with high chemical activity and acid-neutralizing ability. At roasting temperatures below 650 °C, serpentine minerals form a less reactive phase. At temperatures above 750 °C, new crystalline phases with reduced reactivity are formed [10,11].

The roasting procedure is widely used in processing magnesium silicates raw materials, including serpentine minerals [12,13,14,15]. The temperature can affect the phase composition of serpentines, the specific surface area, and porosity [8], which increase the efficiency of the proposed methods.

Performing roasting under optimal temperature conditions is challenging [2] due to the different heat transfer conditions from the furnace to the material for various furnace designs [16]. In particular, roasting parameters on the industrial scale differ significantly from those established in the laboratory [17,18]. Besides, the careful maintenance of the roasting regime in industrial furnaces is needed to save energy resources [17].

Thus, the optimal serpentine roasting mode must be selected for each kiln to obtain a product with the highest acid-neutralizing ability at the lowest cost. It was found for serpentines with high iron content that, as with the high-iron clays, a color change occurs with an increase in the roasting temperature [19,20]. This property can be used to quickly control the properties of the resulting material, depending on its color. The method of comparing materials by color is used, for example, to visually assess the iron content in geological samples of magnesium silicate saprolite [21].

The color characteristic is one of the leading indicators for assessing the resulting product’s properties [22]. Color characteristics, determined by the state and structural features of coloring impurities, can be used to control the compliance of the resulting material characteristics with the specified properties [23].

A photographic image with subsequent processing in a graphics editor is used to obtain the color characteristics of samples. Each color image in virtual space can be described using a color representation algorithm under the general name of the color model, offering a way to describe and obtain colors mathematically. Color models differ in their areas of application [23]. The most common models are RGB (Red, Green, Blue), HSB (Hue, Saturation, and Brightness), and Lab.

In the RGB model, derived colors are obtained by mixing base colors (red, green, blue), which are also called color coordinates [24]. The sum of the 3-color channels gives white [25].

The HSB color model is a nonlinear transformation of the RGB model to the following color coordinates. The hue varies from 0° to 360°, but sometimes it is reduced to a range of 0 to 100 or 0 to 1. Saturation ranges are 0–100 or 0–1. The larger this parameter, the “purer” the color; therefore, this parameter is sometimes referred to as color purity. The closer this parameter is to zero, the closer the color is to neutral gray. Another parameter is brightness, which is also set in the range of 0–100 or 0–1 [26].

The perceived color of an object depends on many factors, such as size, surface texture, and lighting [27]. Human perception of color is a subjective phenomenon that depends on the observer, making it difficult to measure [28]. Almost all color measurement methods are based on the CIE (International Commission on Illumination) specification system to indicate how color can be reproduced [29]. The CIELAB color model has been developed and established as a cross-industry standard in addition to the basic models. The model has a wide color range and is not tied to a light reproduction device. Any color in the model is determined by the value of the brightness of the lightness (L) and two chromatic coordinates, a and b. This model is convenient for characterizing the color of iron-containing pigments [30]. The CIE has suggested using a color difference (∆E) formula that uses objective colorimetry methods and avoids subjective visual assessments [31].

An indicator of ∆E is used to assess the possibility of visually distinguishing similar colors and is defined as the difference between two colors in one of the equal-contrast color spaces. This characteristic considers the difference in the color coordinates L, a, and b of the CIE Lab color space. The Lab characteristic is device-independent, allows an accurate digital assessment, and corresponds to the peculiarities of color perception by the human eye [32]. If the value of ∆E < 2, then it is impossible to distinguish colors by eye, whereas the colors are hardly distinguishable when ∆E is in the range of 2–6. A noticeable difference between the two colors corresponds to ∆E > 6. Thus, we can judge the degree of change in a material’s characteristics by the magnitude of the change in lightness, saturation, and color tone [33,34].

Engineers often face a change in the color characteristics of materials in industries where roasting is used in the technological process. In this case, the evaluation of the roasting conditions and the properties of the obtained materials is possible based on the color of the ultimate product. The goal of this study is to provide a scientific justification for the method of the express control of serpentine thermal activation.

## 2. Materials and Methods

### 2.1. Materials

#### 2.1.1. Characteristics of Initial Materials

Serpentinite magnesite (SM), the overburden of the Khalilovskoye magnesite deposit (Orenburg region, Russia), was used to study the color characteristics of serpentine. The main component of SM is chrysotile; magnesite is contained as an impurity. Two series of thermally activated samples were obtained: samples of the MF series based on chrysotile, manually selected from SM; and RF samples produced by original SM. The chemical composition of both serpentine samples appears in Table 1.

The chemical composition of samples was similar except for MgO, which had a content that was 4% higher in the RF sample due to the magnesite admixture. The high iron content in both samples caused the color change during roasting.

#### 2.1.2. Sample Preparation

Grinding of the serpentine samples was carried out in a ball mill with a mass ratio of material and grinding bodies of 1:3 by weight. The material was milled for 1.5 h, followed by the heat treatment. Material for the MF series was prepared by roasting in a muffle furnace (Nabertherm), sprinkling in a thin layer on a metal pan, and placing that sample in an oven heated to a predetermined temperature (300–850 °C). The roasting time was 20 min.

A laboratory rotary kiln developed, manufactured, and installed at the Institute of Chemistry of the Kola Science Centre of the Russian Academy of Sciences was used to implement the serpentine roasting under nearly industrial conditions. Roasting of RF series samples in the rotary kiln was carried out under the following conditions: a rotation speed of the burning chamber of 40–50 rpm and furnace productivity of 20–23 kg for 9 h. The temperature varied in the range of 300–950 °C by changing the current in the furnace motor’s winding. The heat treatment conditions were controlled by a thermoelectric couple, the loose end of which was placed in the roasting zone’s midsection.

### 2.2. Methods

#### 2.2.1. X-ray Diffraction Analysis

X-ray diffraction (XRD) analysis was performed using a Shimadzu XRD-6000 instrument (Shimadzu Corporation, Kyoto, Japan) with CuKα radiation. Scanning was carried out in a 2θ deg range of 6 to 70° with a step of 0.02° and a dwell time of 1 s. Phases were determined using a PDF-4+2021 program with ICDD © 2021 integrated data-mining software.

#### 2.2.2. Mössbauer Spectroscopy

Mössbauer spectra were recorded on a WISSEL (Starnberg, Germany) spectrometer in the constant acceleration mode at the measurement temperature 300 °C, the source for Co57 measurements in a rhodium (Rh) matrix with an activity of 10 mKi (millicurie). A metal iron absorber that was 15 μm thick was used to calibrate the velocity scale. Isomeric shift (IS) is given relative to αFe.

#### 2.2.3. Optical Absorption Spectroscopy

Optical absorption spectra were recorded on MSFU-K (Lomo, Russia) microscope-spectrophotometer in the wavelength range of 400–800 nm with a step of 1 nm. The device is equipped with a program that allows you to perform mathematical processing of photometry results, including determining the dominant wavelengths; determination of chromatic characteristics in various colorimetric systems (XYZ, CJELAB) is also provided. For an objective measurement and description of color, calculation of chromaticity coordinates according to the XYZ international colorimetric system was performed. All colorimetric results on the interpretation of the optical absorption spectra of minerals were placed on the standard color triangle of the International Lighting Commission of 1964. The colorimetric parameters of the studied minerals according to the international system CIELAB were calculated using the program Spectrum. All experimental studies were carried out at room temperature.

#### 2.2.4. Determination of Serpentines’ Acid-Neutralizing Ability

The serpentine sample’s acid-neutralizing ability was determined by acidimetric titration [35]. The citric acid method of the determination MgO powder reactivity was used [36]. In our work, we used a 0.02 N hydrochloric acid (HCl) solution. A portion of serpentinite (250 mg) was placed in 250 mL of 0.02 N HCl. The suspension was stirred for 3 h and then rested for 24 h for the acid solution’s partial neutralization. Then, the suspension was filtered through a paper filter with a pore diameter of 1–2 μm, and the acid remaining in the solution was titrated with a 0.01 N Na_2_CO_3_; methyl red was used as an indicator. The acid-neutralizing ability (reactivity) of heat-treated serpentines was calculated as the difference between the initial and remaining amounts of acid [9].

#### 2.2.5. Measuring Color Characteristics

Digital images of the samples were obtained using an OSL-528 optical microscope (OSEELANG, ZheJiang, China) combined with a Canon 600D camera (Canon Inc., Tokyo, Japan). Color rendition was calibrated against a color palette and white balance in the Adobe Photoshop Lightroom 5.7.1 64-bit. The resulting color profile was used to correct the digital images of the samples. The color palette, used to adjust the camera’s color profile, was filmed under the same lighting conditions. Correct color digitization of the studied objects required control of the color transfer of the camera under the same lighting conditions. The color characteristics of materials based on the RGB and CIELAB models were determined by averaging the results obtained for three sample images taken in different areas of the sample. Graphic processing was performed using the Adobe Photoshop CC 2015.5.0 software package; data processing was performed using MS Excel 2016.

The inhomogeneity of the sample color due to the nonuniformity of the texture and the presence of impurities was overcome by the digital processing of the original images using the “filter, blur, average” function (Adobe Photoshop CC). This filter determines each of the RGB channels’ average value in the image and assigns it to the image’s entire area. Then, the correction of white balance was performed relative to a photo of white paper, taken under the same conditions. This correction allows researchers to obtain an image most similar to the color perceived by the human eye. Information on the digital characteristics of the sample colors (RGB and Lab) was obtained through the Color Palette panel. The color names and the HEX color codes of the samples were determined using the RGB color model [37].

The ∆E was calculated using the formula approved by CIE:

∆E(Lab) = ((∆L)^2^ +(∆a)^2^ + (∆b)^2^)^1/2^, (1)

where ∆L is the lightness difference between the two samples, ∆a is the difference along the “red (+a)–green (−a) axis”, and ∆b is the difference along the “yellow (+b)–blue (−b)” axis.

Visual assessment of color was carried out in triplicate with a D65 light source (standard daylight). One gram of each sample powder intended for comparison was poured on a glass slide and pressed with a cover glass. The presence or absence of a visual boundary between the powders was determined. Ten researchers were involved in this assessment; inter-subject variability of observations did not occur.

## 3. Results

### 3.1. Influence of the Roasting Temperature on the Phase Composition and Acid-Neutralizing Ability of Serpentinite Magnesite Samples

#### 3.1.1. XRD Analysis

**Figure 1:**
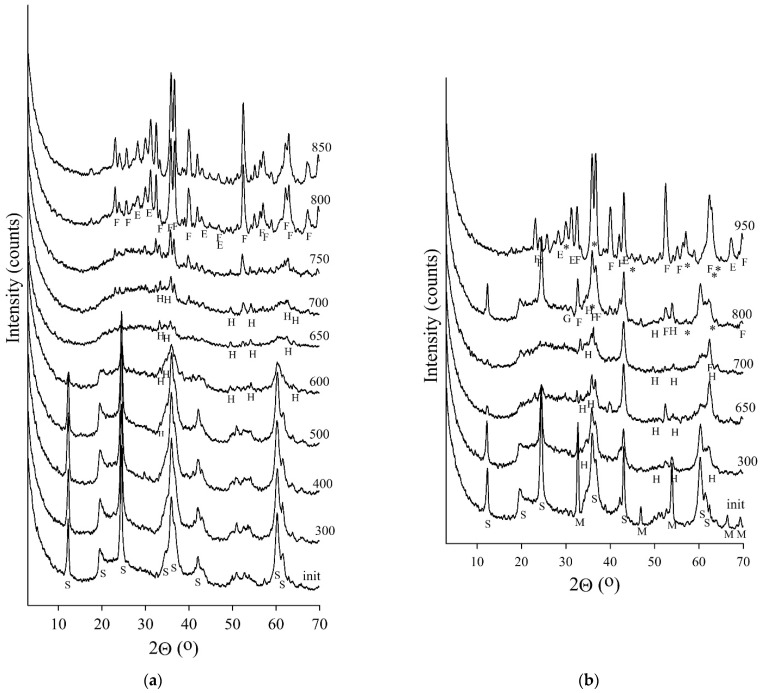
XRD analysis of samples of (**a**)—MF and (**b**)—RF series S-Chrysotile; F—Forsterite; E—Enstatite; H—Hematite; M—Magnesite; *—Magnetite.

Heat treatment of serpentine minerals led to the destruction of the mineral’s crystal lattice and the formation of secondary mineral phases [6] recorded by X-ray phase analysis (Figure 1). As the temperature increased above 500 °C, a decrease in the intensity of the serpentine reflexed, and an increase in forsterite and enstatite was observed while roasting the MF samples in a muffle furnace. It should be noted that at 650 °C, the serpentine reflexes disappeared (Figure 1a).

The XRD results of the RF series samples showed the difference between the serpentine roasting modes in the muffle and laboratory rotary kilns. It is known that the design of the thermal unit has a significant effect on the firing [16]. The processes occurring in two furnaces at the same temperature varied significantly due to the difference in heat transfer conditions from the furnace to the material. This was shown by the data on the phase composition of the MF and RF series samples. For the sample of the MF series roasted at 650 °C, reflections of serpentine were absent, whereas for the RF series, even at temperatures above 800 °C, low-intensity peaks of the initial mineral phase were observed (Figure 1b). During roasting in a muffle furnace, the intensity of forsterite reflections increased at 550 °C, whereas during roasting in a rotary kiln, this took place at a temperature higher than 700 °C.

Because the serpentine color depends both on the amount of impure iron and the changes that occur with the chromophore impurity during heat treatment, it was necessary to study the transformation of iron compounds in the samples with the temperature increase.

According to XRD data, hematite (Fe_2_O_3_) is present in the heat-treated samples. For samples of the MF series, as the roasting temperature increases, the reflections of hematite at 2.696, 2.513, and 1.692 Å become more pronounced than the samples of the RF series. Magnetite reflections appeared in the RF series samples in addition to the hematite reflections when the temperature increased to 800 °C. In the material that was heat-treated at 950 °C, predominantly magnetite reflexes were observed.

Iron hydroxides can transform into hematite Fe_2_O_3_ at 300 ℃ and into magnetite Fe_3_O_4_ at 650 ℃, which again transforms into hematite when cooled under oxidizing conditions [20].

Slow cooling of a large mass (about 10 kg) of material after roasting in a rotary kiln led to the magnetite appearance in the RF series at temperatures above 800 ℃. Similar results were obtained in study of the chrysotile behavior in an electric tube furnace, both ends of which were open to the air [38]. They found that in serpentine heat-treated at temperatures above 700 °C, the magnetite phase appeared according to Mössbauer parameters. Fast cooling of a small portion of serpentine material (70–100 g) in a muffle kiln led to the presence of only the hematite phase in roasted samples.

#### 3.1.2. Mössbauer Spectroscopy

**Figure 2:**
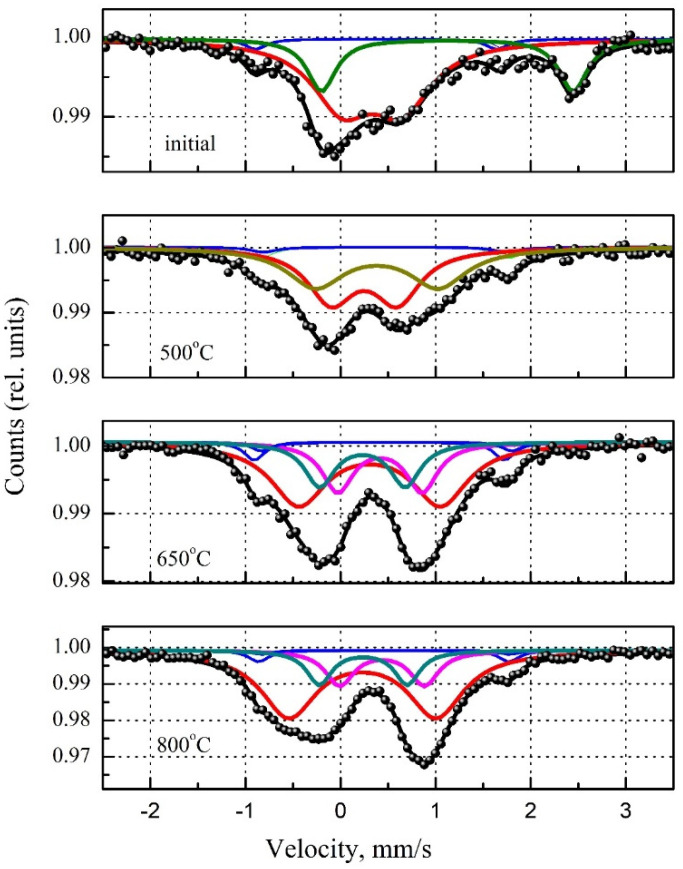
Mössbauer spectra of samples of the MF series.

The Mössbauer spectra of serpentine minerals before and after heat treatment in a muffle furnace are shown in Figure 2. The corresponding Mössbauer parameters are presented in Table 2. In the original serpentine mineral, iron is in four nonequivalent environments. The first two environments are nonmagnetic phases, and their sub-spectra are doublets. Doublet 1 indicates that iron atoms are in the Fe^3+^ state; their proportion is about 49%. The parameters of this doublet are close to the typical Mössbauer parameters for iron in the octahedral position of serpentine minerals (isomeric shift [IS] = 0.332 mm/s, quadrupole splitting [QS] = 0.629 mm/s) [39].

Doublet 2 corresponds to iron atoms Fe^2+^, and their share is about 19%. In doublet 2, the iron parameters are characterized by IS = 1.123 mm/s and QS = 2.646 mm/s. These parameters are in good agreement with the data for iron atoms in the octahedral position of the structure of serpentine minerals from Blaauw et al. (IS = 1.12 mm/s, QS = 2.65 mm/s [39]) and O‘Hanley and Dyar (IS = 1.13 mm/s, QS = 2.71 mm/s [40]).

The two remaining environments correspond to the magnetically ordered state of Fe_2_O_3_ in terms of the Mössbauer parameters. Two sextets with very similar parameters indicated the presence of small amounts of impurities of other elements. The first sextet is almost ideal for standard Fe_2_O_3_. In the second sextet, the underestimated value of H_eff_ often indicates the presence of Al impurities.

In the sample roasted at 500 °C, iron corresponded to two doublets, the parameters of which indicated that the iron atoms were in the Fe^3+^ state but in different crystallographic environments. The doublet corresponding to Fe^2+^ completely disappeared (Figure 2, Table 2). A similar result was obtained by MacKenzie and McGavin [38]. Serpentine heating in air below 200 °C led to a slight change in the Mössbauer spectrum. Fe^2+^ oxidation began at a temperature above 200 °C, interpreted by the Fe^2+^ doublet decrease and octahedral Fe^3^ increase^+^. The oxidation process of iron included in the serpentine structure ends at 500 °C [38].

Three types of Fe^3+^ centers in different crystallographic environments can be distinguished starting from a roasting temperature of 650 °C. This temperature corresponded to the complete disappearance of serpentine reflections. The first doublet was initially presented in the samples, whereas the remaining strongly distorted doublets appeared due to the oxidation of Fe^2+^ and the transition of serpentine mineral to the amorphous state. The serpentine structure’s disordering upon heating was also indicated by an increase in the QS parameter in the first doublet from 0.629 mm/s for the initial sample to 1.549 mm/s for the sample roasted at 800 °C, whereas the IS parameters remained similar (0.22–0.33 mm/s).

In the heat-treated samples, the characteristics of the magnetically ordered phase (Fe_2_O_3_) did not change compared to the initial serpentine mineral. Thus, under the temperature influence, the crystal-chemical state of iron changed only due to the transformation of the serpentine mineral during heat treatment. The optical spectra of the samples can also explain these processes.

#### 3.1.3. Optical Spectroscopy

**Figure 3:**
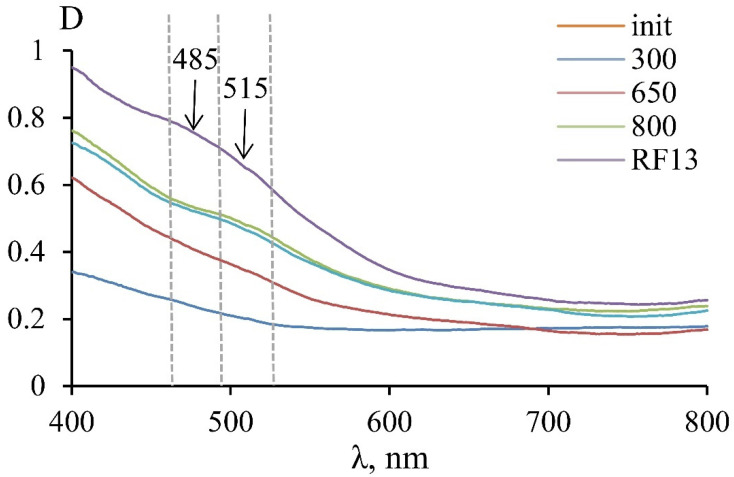
Optical absorption spectrum of MF samples roasted at different temperatures and the RF13 sample roasted at 700 °C.

Studies of the crystal-chemical characteristics and the nature of serpentine color were carried out using optical absorption spectroscopy. The studied samples’ optical spectra were characterized by an intense absorption band in the UV region, which is related to the O^2−^→Fe^3+^ charge transfer mechanism. The color of serpentines was related to the long-wavelength shoulder of this band, which is located in a significant part of the visible spectrum (Figure 3). With an increase in the heating temperature, the total fraction of Fe^3+^ and the absorption in the UV region increased, which caused a rich yellow color of the heat-treated samples.

A monotonic decrease in optical density was observed in the initial sample and the MF sample roasted at 300 °C. A violation of this monotonicity in the 460–525 nm region was observed for the MF samples roasted at 650 and 800 °C and for the RF13 sample. Using spectra mathematical processing, it was found that bands with a wavelength of 515 and 485 nm appeared in this region. The appearance of the 515 nm line in the MF5 sample spectrum, roasted at 650 °C, indicated the spin-forbidden d-d transitions in Fe^3+^ in the octahedral position in the new phase’s structure, which was also detected according to the results of Mössbauer spectroscopy. An additional band at 485 nm was displayed in the spectrum of the sample roasted at 800 °C, which indicates the appearance of a new octahedral position with new linear parameters. According to XRD, the appearance of these lines can be associated with the formation of the serpentine mineral thermal destruction products—amorphous compounds (at 650 °C) and crystalline compounds (at 800 °C).

#### 3.1.4. Activity of MF and RF Series Samples

**Figure 4:**
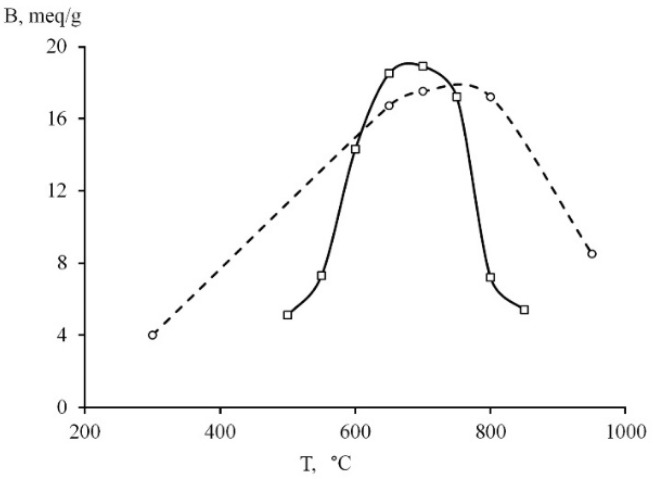
Dependence of the acid-neutralizing ability of serpentine (B, meq/g) on the roasting temperature for the MF series (–□–) and RF series (--○--) samples.

The phase composition change affected the acid-neutralizing ability of serpentine minerals (Figure 4). For the MF series samples, the activity increased at 500–650 °C and reached a maximum at 650–700 °C. Then, a slight decrease in activity occurred at 750 °C, and a sharp decrease occurred at 800 °C. Thus, the optimal temperature for obtaining a thermally activated product using a muffle furnace is in the range of 650–750 °C, which corresponds to data obtained earlier [9]. The sharp increase in the sample’s activity obtained at a temperature of 600 °C should be noted compared to 550 °C, reflecting the beginning of the serpentine destruction already at 600 °C.

The maximal values of the acid-neutralizing ability for the RF series corresponded to the higher roasting temperature in comparison to the MF series. The samples’ activity obtained at 650–700 °C differed insignificantly for both series. At a roasting temperature of 800 °C, the sample activity remained at the maximum level for the RF series and sharply decreased for the MF series. Thus, the results indicated the difference between the roasting processes in the two thermal units. The results of the acid-neutralizing ability of thermally activated serpentine samples obtained in various furnaces showed that the optimal roasting temperature should be set for each heating unit.

**Figure 5:**
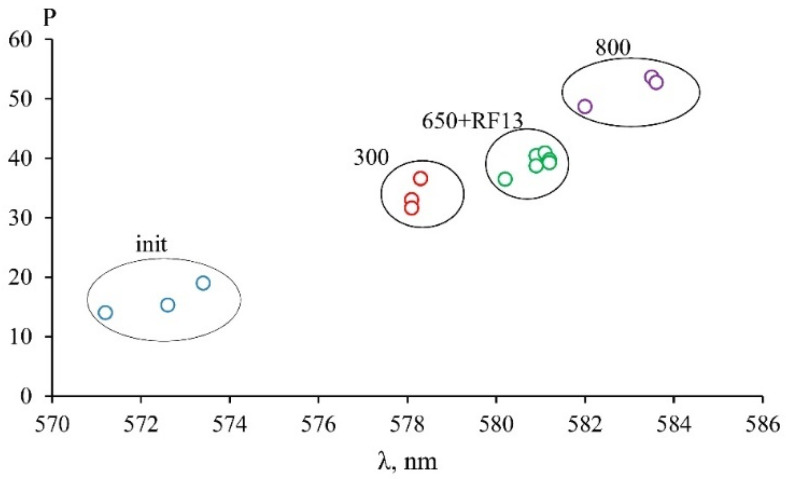
The ratio between the color tone intensity (P) and dominant wavelength (λ) of the MF samples roasted at different temperatures and the RF13 sample roasted at 700 °C.

Under the roasting process, the color of serpentine changed, and the color of the powders became darker with increasing temperature. This property can be used to quickly control the degree of change of the initial serpentine mineral into the active metastable phase. Samples of different series with similar activity values were characterized by similar parameters of the optical spectra (Figure 3, curves 650 and RF13). The results of processing optical spectra with obtaining color parameters in the XYZ color model are shown in Figure 5. The ratio of the color tone intensity and the dominant wavelength for samples of the MF series, roasted at different temperatures, and sample RF13, roasted at 700 °C, showed that samples with a similar activity value also have similar color characteristics obtained by processing optical spectra. Data showed relationships between the activity and the optical spectrum, which, in turn, were determined by the crystal-chemical parameters of iron atoms in the serpentine minerals and the products of their heat treatment. Comparing samples by optical spectra is a time-consuming and expensive process; therefore, visual verification is an acceptable method for expressing the monitoring of serpentine roasting conditions.

### 3.2. Visual Assessment of the Color of Serpentine Sample

**Figure 6:**
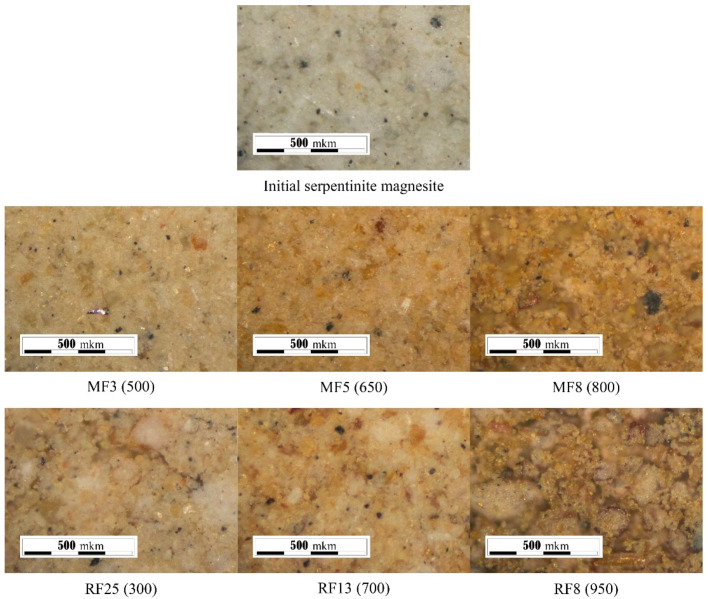
The appearance of serpentine samples obtained in a laboratory muffle furnace (MF) and in a laboratory rotary kiln (RF); the roasting temperature (°C) is indicated in parentheses.

The appearance of the samples of the MF series is shown in Figure 6. The powder of the initial serpentine resembled a uniform gray mass with black particles. Visible red islands appeared in the sample obtained at 500 °C (MF3), in addition to the color change of powder. As the roasting temperature increased, the powder color acquired a bright reddish color with rust-colored particles. Individual particles of samples roasted in a muffle furnace at 800 °C (MF8) appeared caked and dry.

The appearance of serpentine samples of the RF series differed from the samples of the MF series (Figure 6); particles from the RF samples appear larger. Particle enlargement was observed during the roasting of serpentines in the industrial apparatus [41]. The particle size increased due to adhesion during the stirring of the material in a rotating temperature chamber during heat treatment.

The darkest in color sample RF8, obtained at a temperature of 950 °C included a more significant amount of dark gray particles that corresponded to magnetite; the presence of this phase in the samples of the RF series was confirmed by XRD data (Figure 1b).

### 3.3. Determination of the Thermally Activated Serpentine Sample Color Parameters Using Various Models

Firing serpentines led to a change in color characteristics (RGB, Lab). The combination of these characteristics for each obtained sample corresponds to an individual HEX code, which was unique for each sample (Table 3 and Table 4). RGB and HEX color models do not provide an opportunity to numerically display the degree of similarity and difference between the colors of samples. This opportunity is provided by the CIELAB model, namely, ∆E. Colors with low ∆E values can be combined into groups that have a common color name because at ∆E < 2, the ∆E is not perceived by the human eye [33,34].

The coloration of the original sample was close to Desert sand. After heat treatment, the material color changed, and different tones of orange were acquired at different temperatures. A slight color change was observed for samples roasted at temperatures from 300–500 °C; the color of the samples obtained was close to Pale gold. The serpentine sample at roasting temperatures of 650 and 700 °C had an identical color at both temperatures, which was close to Indian yellow. A further increase in temperature up to 750 °C led to material coloration close to Persian orange. At the maximum roasting temperature (900 °C), the serpentine color corresponded to University of California gold.

Comparing the color of materials by name made it possible to distinguish two groups in the temperature ranges of 300–500 °C and 650–700 °C. However, the color name could correspond to a group of colors with different HEX codes, making it challenging to identify the sample’s color. Therefore, an unbiased color comparison should be carried out by the ∆E parameter. The ∆E values were calculated for neighboring pairs of samples. The samples were divided into three groups based on the ∆E parameter, which indicated the ability of the human eye to distinguish the colors.

The results obtained were compared with the acid-neutralizing ability of the samples. For the MF series, the first group included samples with a roasting temperature of less than 650 °C, the second group included samples with maximum activity (obtained in the temperature range of 650–750 °C), and the third group comprised samples with roasting temperatures above 750 °C. Samples of the second group, obtained under optimal conditions, were indistinguishable both in their acid-neutralizing ability and color (∆E 2.2–2.3). The ∆E increased to ∆E 7.7 between samples MF4 and MF5 and ∆E 6.2 between samples MF7 and MF8 when deviating from the heat treatment conditions below (underburning) or above (overburning) the optimal values. Thus, it is possible to determine the conditions for roasting the serpentine samples based on the visual perception of the sample’s color.

The peculiarities of roasting serpentinite magnesite in the rotary kiln were reflected in the color of the obtained samples; their color differed significantly from the samples’ color in the MF series. The representations and color names of the samples of the pilot series are presented in Table 4. Samples RF13, RF18, and RF15 had the same color, Fawen; the color of the RF8 powder was referred to as Café au lait. The data in Table 3 and Table 4 showed that there were practically no coincidences when describing the color of the samples of the RF and MF series using the RGB color model.

∆E values were calculated for the samples of the RF series. The colors of the RF18 and RF13 samples were visually indistinguishable, with a value of ∆E 1. Samples RF13 and RF15 could not be distinguished from each other (∆E 3.3), whereas the ∆E for pairs of samples RF25–RF18 and RF15–RF8 was 14.2 and 15.1, respectively.

Thus, the temperature range of 650–800 °C is optimal for serpentine thermal activation in a laboratory rotary kiln, based on the data for the RF series samples. In this temperature range, practically no change in the color of the material was observed, whereas the deviation of the roasting temperature from the optimal values led to a change in color, which could be reliably established visually.

**Figure 7:**
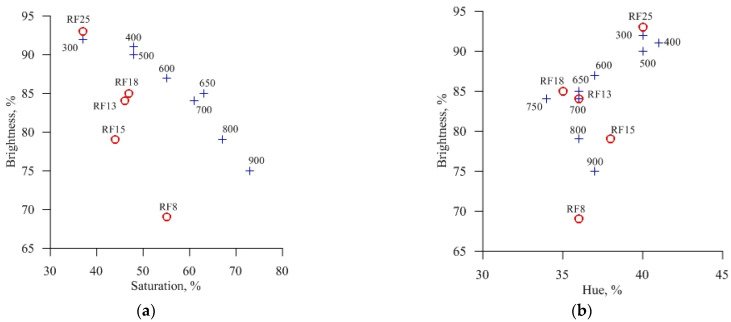
Values of the HSB color model parameters—brightness, saturation (**a**), and brightness, hue (**b**) of the MF (+) and RF (o) samples.

The parameters in the HSB color model are hue, saturation, and brightness. The hue parameter, which characterized the color tone, first decreased with an increase in the roasting temperature and then increased at a temperature above 750 °C (Figure 7b). The color saturation of the MF series samples increased with increasing temperature, whereas the brightness decreased (Figure 7a). The RF series samples showed the same trend; however, the numerical values of the color saturation were lower than those of the MF samples. Compared to the MF series samples, lowering saturation values for the RF series samples, by approximating the color to neutral gray, for example, reflected a higher content of dark gray particles in samples of the RF series.

### 3.4. Express Method of Visual Assessment of the Color of Serpentinite Magnesite

The use of the HSB model allowed researchers to identify the ∆E between the samples roasted at the same temperature but in different furnaces. Based on data from the RGB and CIELAB models, samples of both series with similar values of acid-neutralizing ability did not differ in color. The data on the ∆E parameter, which was calculated using the CIELAB model, indicated that samples with nonoptimal acid-neutralizing ability could be visually distinguished by color. The results allowed us to propose the following algorithm controlling the serpentine roasting.

First, it is necessary to obtain a set of samples by changing the technological parameters of the furnace. For example, for an electric laboratory rotary kiln, these parameters are the voltage applied to the electrical winding and the rotational speed of the kiln. Second, based on the data on the acid-neutralizing ability, it is necessary to determine the optimal range of parameter variation at which the material with the maximum activity can be obtained; then the reference sample is obtained.

Maintaining the selected parameters of the furnace operation in industrial roasting is impossible due to an uncontrolled change in heat transfer conditions from the heated furnace surfaces to the roasted material. Compliance of the roasting parameters with the optimal conditions should be monitored periodically according to the most objective indicator: the properties of the obtained material.

**Figure 8:**
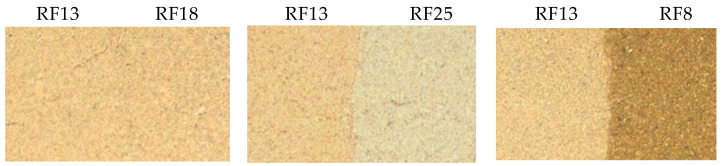
Comparison of the RF samples by color.

Acid-neutralizing ability is the main characteristic of heat-treated serpentine, which correlates with its color. The visual comparison of the color of the resulting material with a reference sample is advisable for the express control of the roasting mode. As an example of such a comparison, Figure 8 shows images of the samples of the RF series. Sample RF13 was selected as a reference. The border between RF13 and RF18 that would indicate similar values for these samples’ activity was invisible. Samples RF25 and RF8 had visible borders with reference sample RF13. The following algorithm could be proposed: RF25 is lighter than the reference RF13 sample; therefore, the roasting temperature should be increased. RF8 is darker than RF13, so the roasting temperature needs to be reduced. The color analysis of the samples should be carried out within approximately one minute, making it possible to adjust the roasting conditions quickly.

## 4. Conclusions

The effect of the roasting temperature on the composition and optical characteristics of the products of the serpentine mineral chrysotile thermal destruction with a high iron content was investigated. Using a set of methods (XRD, Mössbauer spectroscopy, and optical spectroscopy) allowe us to determine that the change in the color of serpentine samples heat-treated under different temperatures was affected by the change in the iron crystal-chemical characteristics. In turn, the crystal-chemical characteristics of iron were determined by the phase composition of the serpentines’ thermolysis products. The phase composition affects the target characteristic of heat-treated serpentine—acid-neutralizing ability. The direct relationship between the optical characteristics of heat-treated serpentine and its activity makes it possible to use the color of the resulting material to control the roasting of serpentines with a high iron content.

This paper systematized the results of determining the color of serpentinite samples with a high iron content obtained at different roasting temperatures in two heating units—a muffle furnace (MF series) and a laboratory electric rotary kiln (RF series). The studies were carried out to substantiate the method for controlling the roasting conditions and the properties of thermally activated serpentines by visually assessing the color of the resulting product. Three color models were used: RGB, CIELAB, and HSB.

Based on the ∆E characteristic (model CIELAB), it was shown that the serpentine samples roasted at the optimal temperature and having the similar values of acid-neutralizing ability did not differ in color. When the roasting mode deviated to higher or lower temperatures, the color of the samples changed; they could be distinguished visually from the material obtained under optimal conditions.

The magnetite formation in the material roasted in the rotary furnace affected the perceived color of the samples. A mismatch in color characteristics of samples obtained in muffle and rotary kilns made it challenging to create a reference palette applied to both heating units. Therefore, it is necessary to create an individual set of samples for different roasting conditions in each furnace.

Based on the results obtained, the following algorithm was proposed for monitoring the conditions of the thermal activation of serpentinite with a high amount of chromophore impurities: (1) obtaining of a series of samples under different roasting parameters (temperature, duration, etc.); (2) determination of the acid-neutralizing ability of the obtained samples; (3) selection of a reference sample with maximum acid-neutralizing ability; (4) establishing of the parameters at which the most active material was obtained for carrying out the technological process of serpentinite roasting; and (5) periodic control of the roasting conditions by visual comparison of the resulting material’s color with the color of the reference sample. In contrast with the determination of acid-neutralizing ability, which takes about one day, the express assessment by color allows the quick quality control of the resulting product.

## Figures and Tables

**Table 1:** Composition of serpentine samples by XRF (%wt).

Sample	MgO	SiO_2_	FeO	Fe_2_O_3_	CaO	Al_2_O_3_	LOI	Impurities
MF	36.3	36.1	1.1	5.4	1.5	1.8	14.6	3.2
RF	40.6	30.9	1.3	6.4	1.3	1.4	15.8	2.3

**Table 2:** Parameters of Mössbauer spectroscopy of the MF series samples.

Temperature, °C	Doublets	IS (mm/s)	QS (mm/s)	%
Initial	1	0.332+/−0.025	0.629+/−0.041	49
	2	1.123+/−0.018	2.646+/−0.036	19
500	1	0.220+/−0.030	0.867+/−0.033	51
	2	0.525+/−0.038	1.176+/−0.085	14
650	1	0.306+/−0.013	1.490+/−0.144	37
	2	0.414+/−0.061	0.887+/−0.067	16
	3	0.231+/−0.060	0.901+/−0.079	14
800	1	0.230+/−0.006	1.549+/−0.028	48
	2	0.437+/−0.025	0.891+/−0.018	13
	3	0.239+/−0.019	0.926+/−0.020	11

**Table 3:** Color characteristics of the samples of the MF series.

Sample	B, meq/g	T, °C	Color Name and Code HEX	Color Model RGB			Color Model CIELAB			
				R	G	B	L	a	b	∆E
MF0	-	Initial	*Desert sand* #E0D0A3	224.0	208.0	163.3	85.0	2.7	26.3	-
				*1.0*	*1.7*	*1.5*	*0.0*	*2.1*	*1.2*	
MF1	-	300	*Pale gold* #EBCE93	234.7	206.3	147.0	85.7	7.7	36.3	11.2
				*0.6*	*0.6*	*1.0*	*0.6*	*0.6*	*0.6*	
MF2	-	400	*Pale gold* #E7C378	231.0	195.0	119.7	82.3	10.7	46.7	11.3
				*1.0*	*1.0*	*1.2*	*0.6*	*0.6*	*0.6*	
MF3	5.1	500	*Pale gold* #E5C177	228.7	193.0	119.3	82.0	11.0	46.0	0.8
				*0.6*	*0.0*	*0.6*	*0.0*	*0.0*	*0.0*	
MF4	14.3	600	*Earth yellow* #DEAF63	222.0	175.3	99.0	77.0	16.7	50.7	8.9
				*0.0*	*0.6*	*1.0*	*0.0*	*0.6*	*0.6*	
MF5	18.5	650	*Indian yellow* #D8A251	216.0	161.7	81.0	73.0	21.0	55.7	7.7
				*1.0*	*0.6*	*1.0*	*0.0*	*1.0*	*0.6*	
MF6	18.9	700	*Indian yellow* #D5A253	213.3	1620.	83.0	72.7	19.7	54.0	2.2
				*0.6*	*1.0*	*1.0*	*0.6*	*0.6*	*1.0*	
MF7	17.2	750	*Persian orange* #D59E54	213.3	158.3	83.7	72.0	21.7	53.0	2.3
				*2.5*	*0.6*	*2.5*	*0.0*	*1.5*	*2.0*	
MF8	7.2	800	*Satin sheen gold* #CA9442	201.7	147.7	65.7	68.0	22.0	57.7	6.2
				*2.1*	*0.5*	*2.1*	*0.0*	*1.2*	*1.6*	
MF9	5.4	900	*University of California gold* #BF8A34	191.0	138.0	52.0	64.0	21.7	60.0	4.6
				*1.7*	*3.6*	*1.7*	*1.0*	*1.5*	*2.0*	

Note: Standard deviations of color models’ coordinates are marked in italics; the column T, °C presented the roasting temperature and color appearance of the samples.

**Table 4:** Color characteristics of the samples of the RF series.

Sample	B, meq/g	T, °C	Color Name and Code HEX	Color Model RGB			Color Model CIELAB			
				R	G	B	L	a	b	∆E
RF25	4.0	300	*Peach-orange* #EDD095	236.7	208.0	149.3	86.0	7.7	35.3	-
				*1.5*	*0.0*	*0.6*	*0.0*	*0.6*	*1.2*	
RF18	16.7	650	*Fawen* #DAAF73	217.7	174.7	114.7	76.0	16.0	41.0	14.2
				*0.6*	*0.6*	*0.6*	*0.0*	*0.0*	*0.0*	
RF13	17.5	700	*Fawen* #D8AD71	215.7	173.3	113.3	76.0	14.7	40.7	1.4
				*0.6*	*0.6*	*0.6*	*1.0*	*1.5*	*0.6*	
RF15	17.2	800	*Fawen* #D5B173	213.3	176.7	115.3	75.3	11.7	39.3	3.3
				*1.5*	*3.5*	*2.5*	*2.1*	*0.6*	*2.1*	
RF8	8.5	950	*Café au lait* #B2874B	178.0	135.3	75.3	62.0	17.0	44.0	15.1
				*2.0*	*0.6*	*1.5*	*0.0*	*1.0*	*2.0*	

Note: Standard deviations of color models’ coordinates are marked in italics; the column T, °C presented the roasting temperature and color appearance of the samples.

## Data Availability

Data Sharing is not applicable.
